# First episode psychosis related to COVID-19 infection

**DOI:** 10.1192/j.eurpsy.2022.1349

**Published:** 2022-09-01

**Authors:** S. Dhakouani, R. Kammoun, M. Skini, M. Karoui, F. Ellouz

**Affiliations:** 1 Razi hospital, G, Tunis, Tunisia; 2 Razi Hospital, Psychiatry G, manouba, Tunisia

**Keywords:** Neuropsychiatry, Psychosis, covid 19

## Abstract

**Introduction:**

During the course of COVID-19 pandemic, The respiratory system is the most commonly affected while many neuropsychiatric manifestations of the disease have been observed.

**Objectives:**

Emphasize the importance of eliminating the diagnosis of covid 19 infection in a pandemic context face to first episode psychosis.

**Methods:**

Presentation of case report

**Results:**

A 29-year-old woman unemployed married with no personal medical history and with psychiatric family history. She wasn’t exposed to subject with covid 19 in her family circle. She was admitted in psychiatric care for acute behavioural disorders during five days. On physical examination: she was afebrile, eupneic and tachycardiac. Oxygen saturation was 96% and blood pressure was 100/50 mmHg. Specialized neurological examination was normal and cerebral CT scan was without abnormalities. At the psychiatric interview she was extremely agitated. She was distressed her speech was incoherent. She had auditory and visual hallucinations and a multi-thematic delirium. One day after her admission she died suddenly, the autopsy found positive RT PCR covid test and bilateral basal pneumonia.

**Conclusions:**

In individuals presenting with new-onset psychosis in areas endemic to COVID-19, consideration should be made for neuropsychiatric manifestations of Covid 19 from where the importance to push the explorations and to test the patients.

**Disclosure:**

No significant relationships.

